# Storage stability of non-encapsulated pneumococci in saliva is dependent on null-capsule clade, with strains carrying aliC and aliD showing a competitive disadvantage during culture enrichment

**DOI:** 10.1099/mic.0.001585

**Published:** 2025-08-01

**Authors:** Claire S. Laxton, Orchid M. Allicock, Chikondi Peno, Tzu-Yi Lin, Alidia M. Koelewijn, Femke L. Toekiran, Luna Aguilar, Anna York, Anne L. Wyllie

**Affiliations:** 1 Department of Epidemiology of Microbial Diseases, Yale School of Public Health, New Haven, CT, USA

**Keywords:** carriage, mitis-group streptococci, non-encapsulated pneumococcus, qPCR, saliva, stability

## Abstract

Non-encapsulated *Streptococcus pneumoniae* (NESp) represent up to 19% of circulating pneumococci and exhibit high rates of genetic exchange and antimicrobial resistance. Saliva is increasingly used as a pneumococcal carriage study specimen, and we recently developed a qPCR assay to enhance carriage surveillance and characterization of NESp in saliva. Previous work has established that pneumococci remain viable in unsupplemented saliva for extended periods under various conditions. However, these findings may not be applicable to NESp. Therefore, to ensure the robustness of NESp detection in saliva-based carriage studies, we evaluated the impact of transport and storage conditions of saliva samples on NESp detection. Six NESp strains from two clinically relevant NESp null-capsule clades (NCCs), NCC1 (carrying *pspK*) and NCC2 (carrying *aliC* and *aliD*), were spiked into pneumococcus (*lytA*)-negative saliva and incubated through various temperatures and freeze-thaw conditions. Endpoints were processed using either culture enrichment (CE) and DNA extraction (CE-DNA), or an extraction-free method without CE, before testing for *lytA* using qPCR. Detection stability was assessed using linear regression modelling over temperature, time and freeze-thaws. Following CE-DNA, detection of NESp remained stable for ≤24 or ≤72 h when stored at room temperature or 4 °C, respectively, and over two freeze-thaw cycles (−80 °C), with glycerol supplementation providing slight benefits. Stability of detection when using CE-DNA depended on NCC; detection of NCC2 strains was lower and less stable than NCC1. Compared to CE-DNA, extraction-free detection was more stable, with no significant loss over 72 h at room temperature and over three freeze-thaw cycles, and negligible differences in detection between NCC1 and NCC2 strains. Additionally, extraction-free detection of NCC1, and less so NCC2, increased over the first 24 h when stored at 20–30 °C, suggesting growth of the NESp strains in saliva. Testing of *ΔaliCaliD* and *ΔpspK* mutants revealed that these genes increased *in vitro* viability when cultured in broth but did not significantly alter competitive fitness during saliva CE. The NCC1 NESp strains tested exhibited similar stability patterns in unsupplemented saliva as encapsulated pneumococci. However, the NCC2 strains tested here were less resilient during CE, likely due to competition with other oral microbes. Therefore, recovery of NCC2 NESp may be impacted by transport and storage conditions, leading to an underestimation of carriage prevalence when tested using CE-based methods. For the reliable carriage surveillance of NESp, samples should be stored at 4 °C soon after collection and at −80 °C within 72 h. Methods which directly detect DNA without CE may provide a less biassed accounting of NCC2 strains.

## Introduction

The upper respiratory tract commensal bacterium, *Streptococcus pneumoniae* (pneumococcus), remains the leading global cause of morbidity and mortality from lower respiratory infections. The introduction of pneumococcal conjugate vaccines (PCVs) has led to a substantial reduction in pneumococcal disease caused by up to 21 of the 100+ pneumococcal serotypes described to date [1,2]. Concomitantly, PCV use has driven shifts in pneumococcal population dynamics, leading to increased prevalence of non-vaccine-targeted serotypes and non-encapsulated *Streptococcus pneumoniae* (NESp), which lack a polysaccharide capsule [3,6]. Carriage prevalence of NESp accounts for 3–19% of circulating pneumococcal strains [7,8]. This is of particular concern due to their heightened propensity for genetic exchange, including of antibiotic resistance elements, leading to high rates of multidrug resistance among NESp strains [9,17]. It is therefore important that active surveillance of the pneumococcal landscape includes both typable and non-typable pneumococcal strains.

NESp are divided into two groups: group I, which retains non-functional capsular genes, and group II, which completely lacks capsular genes in the *cps* locus [18]. Group II NESp are further classified into NCCs based on the presence of specific genetic elements within the cps locus, including *pspK* (NCC1), *aliC* and *aliD* (NCC2, referred to as Swiss_NT by SeroBA 1.0 [19]), only *aliD* (historically NCC3) or transposable elements alone (NCC4) [7,20,22]. The genes present in NCC1 and NCC2 strains, *pspK*, *aliC* and *aliD*, are virulence factors which can compensate for the lack of polysaccharide capsule. AliC and AliD regulate the expression of choline-binding protein AC (CbpAC), which helps reduce C3b deposition on the bacterial surface, thereby enhancing resistance to classical complement-mediated clearance [23,24]. PspK increases biofilm formation and bacterial adherence to epithelial cells. In turn, this reduces secretory IgA-mediated nasopharyngeal clearance and enhances transmission during influenza co-infection [12,20, 25].

Extensive research has been conducted on encapsulated pneumococci due to the critical role of the capsule in strain virulence. While the absence of capsule generally reduces virulence, NCC1 and NCC2 strains have been associated with conjunctivitis outbreaks, otitis media and occasional cases of invasive pneumococcal diseases [7,14, 26,29]. Pneumococcal carriage studies often neglect to further classify serologically non-typable strains; thus, the prevalence of NCC1 and NCC2 NESp is most likely underreported [5,7, 13, 30]. We recently sought to remedy this by developing a multiplex-qPCR method to simplify the classification of group II NESp and increase the inclusion of NESp monitoring in future carriage studies [31].

While nasopharyngeal swabs remain the gold-standard sample for pneumococcal carriage detection [32], swab collection is invasive and resource-intensive, requiring specialized materials and trained healthcare professionals. Saliva is a more accessible sample type than swabs [33] and is increasingly being used for pneumococcal carriage detection [34,39]. As such, the use of saliva for community-based studies continues to be validated and optimized. For example, we and others have demonstrated the high storage stability of respiratory pathogens, including encapsulated pneumococci, in unsupplemented saliva samples [40,43]. However, the stability of NESp, particularly the potentially more virulent NCC1 and NCC2 strains, has not yet been similarly investigated. Therefore, we examined the stability of the detection of NESp in saliva, over different temperatures, timespans and freeze-thaw cycles, to better inform the experimental design of future saliva-based pneumococcal carriage studies and improve the accuracy of NESp carriage estimates.

## Methods

### Saliva samples

Participants were asked to provide whole-mouth unstimulated saliva by passively drooling saliva into a sterile 25 ml polypropylene tube at least 30 min after eating, drinking or brushing their teeth. Samples were transferred at room temperature to the laboratory within 30 min of collection for temporary storage at 4 °C, and aliquots were made and stored at −80 °C within 12 h.

Each sample was thoroughly screened for the absence of pneumococci by incubating an aliquot of each sample at 37 °C and tested at 24, 48 and 72 h for the genes encoding the major pneumococcal autolysin LytA (*lytA*) [44] and the pneumococcal iron uptake ABC transporter lipoprotein PiaB (*piaB*) [45,46], using methods described previously [31]. Saliva samples which tested negative for both *piaB* and *lytA* were thawed, pooled, aliquoted and either placed on ice for immediate use or stored until needed at −80 °C.

### Bacterial isolates

Pneumococcal isolates (Table 1) representing the major NCCs (NCC1, 2a and 2b) were obtained from Lance Keller and Larry McDaniel (University of Mississippi Medical Centre, USA), Moon H Nahm (University of Alabama, USA) and Ron Dagan (Ben-Gurion University, Israel) [29,47, 48].

**Table 1.** Strains of NESp used in this study

Strain	NCC, MLST	Source	Whole-genome sequence accession
MNZ41	NCC2b, 6153	Carriage isolate, South Korea [48]	ASJQ00000000 [58]
MNZ11	NCC1, 6151	Carriage isolate, South Korea [48]	ASJW00000000 [58]
MNZ85	NCC2a, 2315	Carriage isolate, South Korea [48]	ASJF000030000 [58]
C144.66	NCC1, 9570	Adenoiditis isolate, USA [29]	LSMB00000000.1 [29]
D37	NCC2, NF	Carriage isolate, Israel [47]	JBJLPC000000000 [31]
D48	NCC2, NF	Carriage isolate, Israel [47]	JBJLPD000000000 [31]
MNZ1131	NCC1, 6151	MNZ11 *ΔpspK* mutant [48]	–
JLB01	NCC2b, 6153	MNZ41 *ΔaliCaliD* mutant [24]	–

NF, MLST not found.

Isolates were plated as a lawn onto tryptic soy agar II plus 5% (v/v) defibrinated sheep blood (blood plates) and incubated at 37 °C with 5% CO_2_ overnight. The lawn was harvested into 1 ml brain heart infusion (BHI) medium using a cotton swab and used to inoculate 45 ml BHI medium. Cultures were grown at 37 °C with 5% CO_2_ to an OD at 620 nm (OD_620_) of ~0.6 absorbance units. Cultures were harvested by centrifugation at 4,000 ***g***, and pellets were resuspended in 5–10 ml BHI medium supplemented with 10% (v/v) glycerol and stored at −80 °C. The bacterial concentration (c.f.u. per millilitre) of each sample was determined by colony counting of serially diluted samples cultured on blood plates and incubated at 37 °C with 5% CO_2_ overnight. Preliminary work from this study (data not shown) and other studies has found that due to aggregate formation, vigorous vortexing is required between serial dilution of NESp [49]. The recommended approach, to dilute into individual 1.5 ml microcentrifuge tubes with vortexing for 5–10 s between dilutions, was adopted here to ensure accurate colony counting.

### Experimental design

For each isolate listed in Table 1, concentrated stocks were diluted in BHI and then spiked into saliva at final concentrations of 1,000 and 10,000 c.f.u. ml^−1^ (final concentration of BHI<3% v/v), as described previously [40]. Spiked saliva samples were incubated at 4 °C, room temperature (~20 °C) and 30 °C. At 24 h, 48 h and 72 h, the samples were vortexed, and 100 µl was removed from each for culture enrichment (CE) (plated immediately, timepoint 0) to test for strain viability as described previously [31,40]. A further 50 µl of each sample was aliquoted and stored at −80 °C for extraction-free pneumococcal detection [43].

To determine the effect of freeze-thawing on the stability of pneumococcal detection, saliva was spiked with pneumococci to final concentrations of 1,000 and 10,000 c.f.u. ml^−1^ as above (unsupplemented) or supplemented with BHI with 50% glycerol (final concentrations of 30% BHI v/v and 15% glycerol v/v). Aliquots were stored for a minimum of 3 h at either −20 °C or −80 °C to allow complete freezing, before being thawed at room temperature. Once thawed, the samples were vortexed, and 100 µl was removed from each sample for CE before the remainder was returned to −20 °C or −80 °C. This process was repeated twice more. Where sample volume was insufficient for processing due to technical issues, the datapoint was excluded.

To further examine the recovery of strains immediately following spiking, representative NCC1 (MNZ11) and NCC2 (MNZ41) strains were spiked at 15,000 c.f.u. ml^−1^ into five different *lytA*- and *piaB*-negative saliva samples collected and prepared as above. Each individual saliva sample was spiked in a matched pairs fashion. Stains were spiked into 1 ml BHI in the same way in triplicate. Following spiking, samples were vortexed for 10 s and then plated for CE within 10 min.

### Saliva sample processing by CE-DNA extraction or extraction-free methods

Culture-enriched saliva samples were thawed at room temperature, and DNA was extracted from 200 µl of each sample using the MagMAX Viral/Pathogen Nucleic Acid Isolation Kit (MVP I) using a KingFisher Apex instrument (Thermo Fisher Scientific), with modifications [39]. The method is referred to here as CE-DNA extraction.

Spiked saliva samples were additionally processed using an extraction-free method, without CE, as described previously [43]. This method involves a lysis step with proteinase K, heat inactivation at 95 °C for 10 min and immediate transfer of the treated sample (which we will refer to as lysate) for pneumococcal detection via qPCR.

### Detection of pneumococcal carriage

Each sample (2.5 µl of either DNA template or extraction-free lysate) was tested using the same dualplex qPCR targeting *piaB* and *lytA* as described above. Genomic DNA extracted from a pneumococcus serotype 19A strain (*lytA*- and *piaB*-positive) was included in every plate as a positive control, using at least two standard concentrations (0.0001–1 ng µl^−1^). Assays were run on a CFX96 Touch instrument (Bio-Rad) under the following conditions: 95 °C for 3 min followed by 45 cycles of 98 °C for 15 s and 60 °C for 30 s. Samples were considered positive for pneumococci when the quantification cycle (Cq) values for both genes were ≤40 and within 2 Cq of each other, and negative controls were undetectable [50]. Since NESp strains MNZ41, C144.66 and D48 are naturally *piaB*-negative, only *lytA* positivity was considered for samples spiked with these strains [31]. Plate-to-plate variation was corrected for by multiplying each sample Cq by an adjustment factor as described previously [31].

### Statistical analyses

Linear regression using R (version 3.5.2) was conducted to evaluate the impacts of time and temperature (time) or freeze-thaw cycle and supplementation (freeze-thaw) on the detection of pneumococci from spiked saliva samples using the *lytA*-adjusted Cq. Interaction terms were used to evaluate whether the effects of time and temperature (time), freeze-thaw cycle and supplementation (freeze-thaw) and starting concentration (both) varied by strain. The ΔCq value represents the change in the mean Cq value from freshly spiked saliva under each condition (categorical). *P* values of less than 0.05 were considered significant. Tabulated model results are available in the Supplementary Material.

To further study viability following CE-DNA extraction of NCC1 vs NCC2 strains immediately after spiking into saliva or BHI, a mixed-effects analysis was employed using GraphPad Prism (version 10.3.0) with Tukey’s multiple comparisons, utilizing a restricted maximum likelihood algorithm due to differently sized groups. The experiment was conducted in a repeated measures fashion, and the ΔCq value represents the change in the mean value between each strain. *P* values of less than 0.05 were considered significant.

## Results

### Detection of culture-enriched NESp depends on storage conditions, supplementation and NCC

**Fig. 1.**
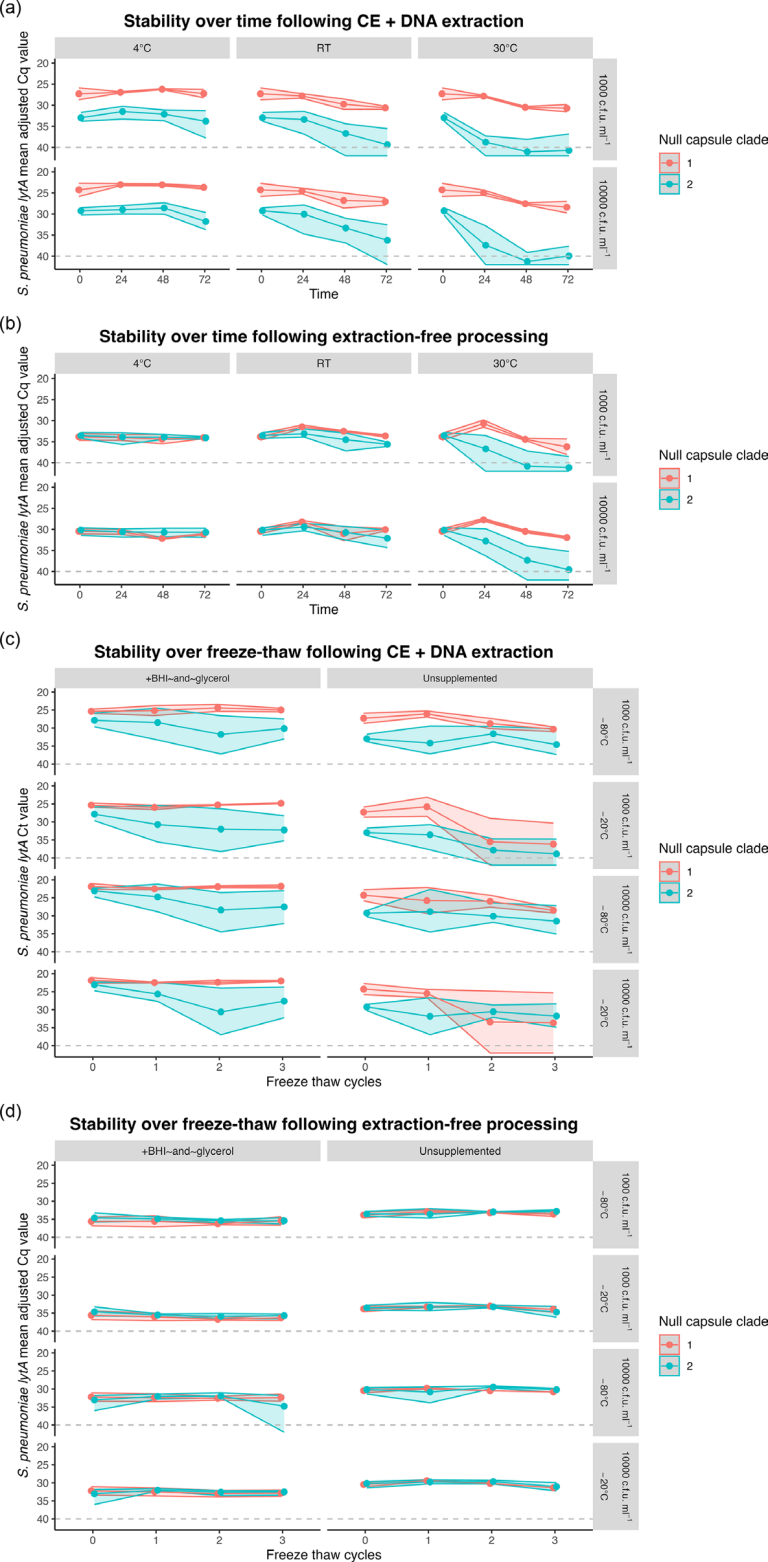
Detection of NCC1 NESp over time, temperature and freeze-thaw is more stable than that of NCC2 when spiked into saliva. Panels (a) and (b) show the detection of NESp over time at different temperatures (4 °C, RT=room temperature and 30 °C) following either culture enrichment and DNA extraction (CE+DNA) or extraction-free methods, respectively. Pooled saliva was initially spiked at either 1,000 or 10,000 c.f.u. ml^−1^, as indicated by the right-hand label. Panels (c) and (d) show the detection of NESp over three freeze-thaw cycles following either CE+DNA or extraction-free methods, respectively. Pooled saliva was initially spiked into unsupplemented saliva or saliva supplemented with BHI plus glycerol at either 1,000 or 10,000 c.f.u. ml^−1^ and cycled through either −20 °C or −80 °C, as indicated by the right-hand label. Data are shown as the mean adjusted Cq for *lytA* for each of NCC1 (*n*=2, red) or NCC2 (*n*=4, blue) with the range shown as a shaded ribbon.

When tested following CE-DNA extraction, the stability of detection of NESp over time was dependent upon temperature (Fig. 1a). There was no significant loss in detection over 72 h when stored at 4 °C (ΔCq=1.01, *P*=0.083, 95% CI: −0.14 to 2.16). However, detection dropped significantly after 48 h when stored at RT (ΔCq=3.44, *P*<0.001, 95% CI: 1.50–5.38), and after 24 h when stored at 30 °C (ΔCq=4.86, *P*<0.001, 95% CI: 2.76–6.97). In addition, the stability of detection over time and temperature depended on NCC. When stratified by NCC, detection of NCC2 strains, but not NCC1, dropped significantly after 72 h when stored at 4 °C (ΔCq=1.68, *P*=0.035, 95% CI: 0.12–3.24), after 48 h when stored at RT (ΔCq=3.92, *P*=0.006, 95% CI: 1.20–6.64), and for less than 24 h when stored at 30 °C (ΔCq=7.00, *P*<0.001, 95% CI: 4.38–9.60).

We next assessed the effect of freeze-thaw cycles on the detection stability of NESp (Fig. 1c). For samples that were processed by CE-DNA extraction, detection stability of NESp across freeze-thaw cycles was dependent on storage temperature. There was a significant reduction in detection by the second freeze-thaw cycle for samples stored at −20 °C (ΔCq=4.46, *P*<0.001, 95% CI: 2.25–6.68) and by the third cycle for samples stored at −80 °C (ΔCq=2.34, *P*=0.008, 95% CI: 2.25–6.68), suggesting a slightly greater freeze-thaw stability when stored at −80 °C.

When tested using CE-DNA extraction, adding BHI+glycerol to the saliva significantly increased overall bacterial detection following freeze-thaws at −80 °C (ΔCq unsupplemented vs glycerol=3.79, *P*<0.001, 95% CI: 2.58–5.00) and −20 °C (ΔCq unsupplemented vs glycerol=5.22, *P*<0.001, 95% CI: 3.65–6.78); however, glycerol supplementation did not affect the stability of detection through consecutive freeze-thaws (ΔCq 0 h vs 72 h=1.62, *P*=0.251, 95% CI: −1.16 to 4.41) (Fig. 1c). Similarly, while overall detection of NCC2 was lower than that of NCC1 strains (ΔCq=4.20, *P*<0.001, 95% CI: 3.17–5.25), stability of detection across freeze-thaw cycles was unaffected by NCC (ΔCq=0.42, *P*=0.781, 95% CI: −2.56 to 3.40).

### Processing of NESp using an extraction-free method leads to greater stability of detection which is less dependent on storage conditions and NCC

To better understand the impact of culture enrichment on the detection of NESp strains, spiked saliva samples which underwent the same storage conditions were also tested using the extraction-free method, which does not include CE. Similarly to when tested using CE-DNA extraction, detection was stable over 72 h when stored at 4 °C (ΔCq=0.50, *P*=0.127, 95% CI: −0.15 to 1.15). However, when stored at room temperature, there was a slight increase in overall detection at 24 h (ΔCq=−1.14, *P*=0.017, 95% CI: −2.07 to −0.21), which became a decrease in detection by 72 h (ΔCq=1.24, *P*=0.10, 95% CI: 0.31–2.16), suggesting that some strains may have grown a small amount in the saliva. At 30 °C, overall detection dropped after 48 h (ΔCq=4.91, *P*<0.001, 95% CI: 2.86–6.97).

Unlike with CE-DNA extraction, there was no difference in stability of detection of NCC at either 4 °C or room temperature when processed using the extraction-free method (Fig. 1b). Detection according to NCC only deviated when stored at 30 °C, in which detection of NCC2 strains decreased significantly after 24 h (ΔCq=2.88, *P*<0.027, 95% CI: 0.35–5.40), whereas detection of NCC1 strains, like at room temperature, increased at 24 h (ΔCq=−2.93, *P*<0.002, 95% CI: −4.55 to 1.30), before decreasing by 72 h (ΔCq=1.92, *P*<0.025, 95% CI: 0.29–3.54). This suggests that NCC1 strains, and less so NCC2 strains, can grow in whole saliva at temperatures between 20 and 30 °C.

Detection of NESp remained stable over all three freeze-thaw cycles when processed using the extraction free method (ΔCq=0.47, *P*=0.058, 95% CI: −0.02 to 0.95), and we did not observe any impact on NESp detection over repeated freeze-thaw cycle by NCC strain (ΔCq=−0.14, *P*=0.470, 95% CI: −0.51 to 0.23), or storage temperature (ΔCq=−0.12, *P*=0.507, 95% CI: −0.23 to 0.47) (Fig. 1d). Unexpectedly, addition of glycerol supplementation resulted in slightly lower overall detection when samples were processed using the extraction-free method (ΔCq unsupplemented vs supplemented=−2.22, *P*<0.001, 95% CI: 2.57–1.87). Glycerol is typically considered a qPCR enhancer and cryoprotectant [51]; however, it is possible that the addition of glycerol in this concentration or formulation reduced the efficiency of the proteinase K or heat lysis step, resulting in less DNA available for qPCR.

Overall, there were twice as many samples in which *lytA* was not detectable at the endpoint following CE-DNA extraction (7.4%) than extraction-free processing (3.4%) (Table 2). Almost all the samples in which *lytA* could not be detected (32 out of 663) were originally spiked with an NCC2 strain, except for four unsupplemented saliva samples which were spiked with an NCC1 strain and underwent ≥2 freeze-thaw cycles.

**Table 2.** Percentage of samples from which NESp was not detectable (ND) stratified by treatment group

Processing method	Strain	Storage conditions* (% ND)	Freeze-thaw† (% ND)		Total (% ND)
			Unsupplemented	+BHI and glycerol	
CE and DNA extraction	**NCC1**	0/48 (0.0%)	4/32 (12.5%)	0/32 (0.0%)	4/112 (3.6%)
	**NCC2**	18/96 (18.8%)	3/64 (4.7%)	0/64 (0.0%)	21/224 (9.4%)
Extraction-free	**NCC1**	0/48 (0.0%)	0/28 (0.0%)	0/32 (0.0%)	0/108 (0.0%)
	**NCC2**	10/96 (10.4%)	0/59 (0.0%)	1/64 (1.6%)	11/219 (5.0%)
Total (% ND)		28/288 (9.7%)	7/183 (3.8%)	1/192 (0.5%)	36/663 (5.4%)

*All samples included in Fig. 1(a, b), stored at various temperatures over time.

†All samples included in Fig. 1(c, d) subjected to varying cycles of freeze-thawing.

**Fig. 2.**
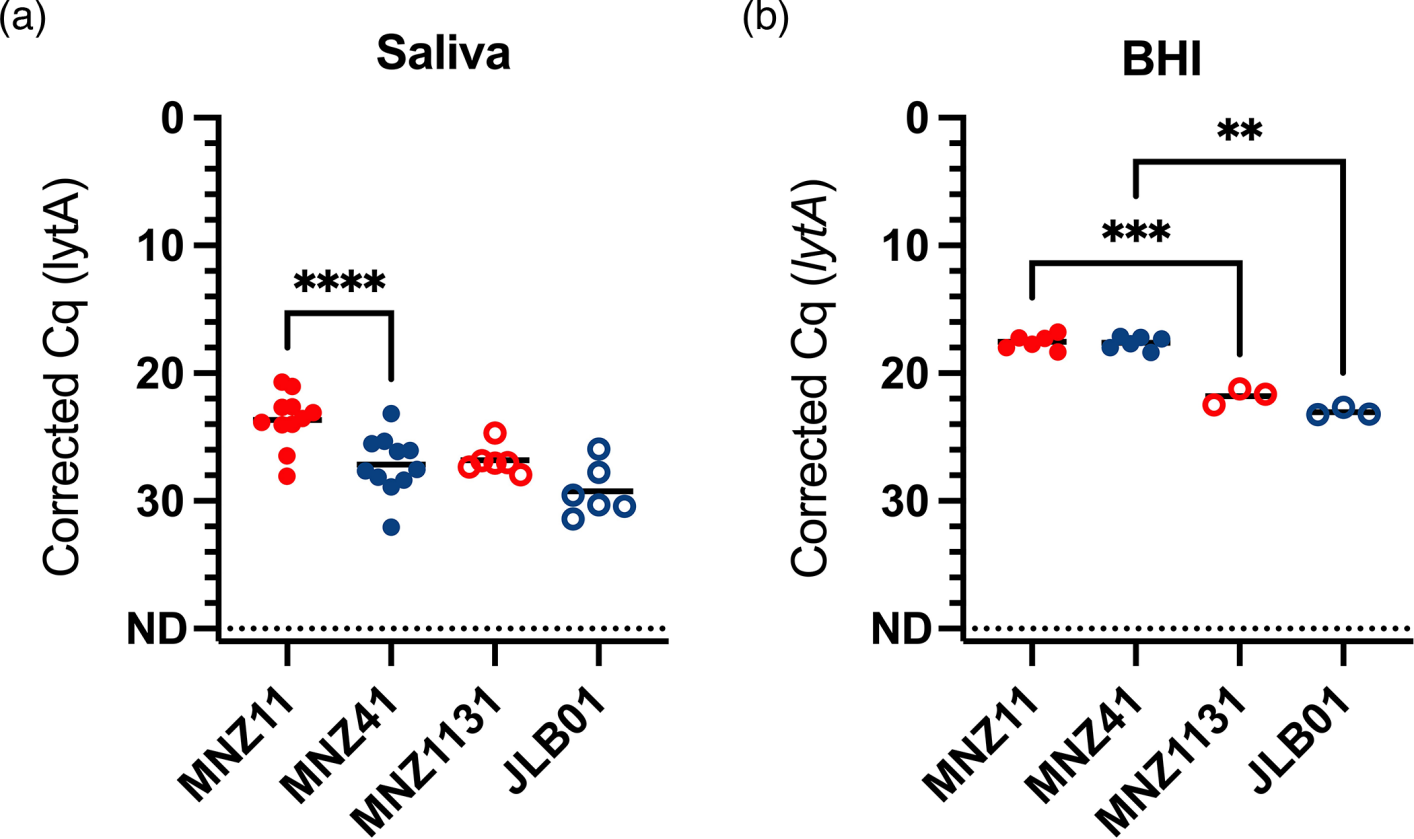
Recovery of NCC2 NESp is lower than NCC1 immediately after spiking into saliva, but not BHI, and this is not dependent on *aliC* and *aliD*, or *pspK*. MNZ11 (red, filled circles) is NCC1, MNZ41 (blue, filled circles) is NCC2 and MNZ1131 (red, open circles) and JLB01 (blue, open circles) are *pspK* and *aliC*/*aliD* gene knockouts of MNZ11 and MNZ41, respectively. Strains were spiked at a final concentration of 15,000 c.f.u. ml^−1^ into either BHI (*n*=6 for MNZ11 and MNZ41, *n*=3 for MNZ1131 and JLB01) or matched pairs of *lytA*- and *piaB*-negative saliva from healthy individuals (*n*=11 for MNZ11 and MNZ41, *n*=6 for MNZ1131 and JLB01). Line is the mean, and significance is shown for multiple comparisons between MNZ11 vs MNZ41, MNZ11 vs MNZ1131 and MNZ41 vs JLB01, **P<0.005, ***P<0.0005, ****P<0.0001.

Following the observation that NCC2 strains were less stable in saliva than NCC1 strains following CE, we sought to determine if this was driven by the presence of *aliC* and *aliD*. Strains belonging to NCC1 (MNZ11) and NCC2 (MNZ41) were spiked into saliva and BHI in parallel and immediately tested using the CE-DNA extraction method. Recovery of viable NCC1 strains was significantly higher than NCC2 following spiking into saliva (mean ΔCq=−3.52, 95% CI: −4.67 to −2.37, *P*<0.0001; Fig. 2a). However, there was no significant difference when spiked into BHI (mean ΔCq=−0.06, 95% CI: −1.19 to 1.08, *P*=0.9972; Fig. 2b). Recovery of both strains was lower and more varied in saliva than in BHI, suggesting that a component of saliva has an impact on bacterial viability during CE, which was more pronounced for NCC2 strains.

Knockout mutants MNZ1131 (*ΔpspK*) and JLB01 (*ΔaliCaliD*) were also tested, and while recovery in BHI was significantly lower for both when compared to their wild types (Fig. 2b), there were no significant differences in recovery from saliva between the respective wild types and mutants. This suggests that while *pspK*, *aliC* and *aliD* do play a role in overall cell fitness, they are not primarily driving the effect on viability of NESp during CE in saliva.

## Discussion

Carriage studies are reporting general increases in the prevalence of NESp, which is of concern, particularly in the case of NCC1 and NCC2 strains, due to the increased virulence of these strains compared with other NESp, and their propensity to acquire AMR genes [5,7, 13, 15, 16]. This is particularly true for *pspK+* strains, which have some of the highest frequencies of genetic exchange among pneumococci [17]. There has also been an increase in recent years in the use of saliva as a sample type for investigating pneumococcal carriage; however, the impact of saliva sample storage on the detection and recovery of NESp has not yet been described [34,36, 38, 45, 52,54].

Therefore, in this study, we sought to build upon the existing evidence examining encapsulated pneumococci viability in saliva across a range of storage conditions and determine if similar findings applied to NESp. We previously demonstrated that the viability of pneumococci remained stable in self-collected, unsupplemented saliva samples stored between 4 and 30 °C for up to 24 h, and a portion of viable pneumococci could be detected for up to 72 h, though at lower quantities [40]. Findings from this current study demonstrate that while the NCC1 strains tested here exhibited equivalent storage stability in saliva to encapsulated strains [40], the NCC2 strains tested were less able to survive CE, leading to lower detection than NCC1 following CE-DNA extraction (Figs 1a, c and 2a).

Overall, NESp detection using an extraction-free method was generally lower compared with detection following CE-DNA extraction. This finding was expected, due to the lack of enrichment and DNA concentration steps in the extraction-free method. Interestingly, though, compared with CE-DNA extraction, the extraction-free method yielded more stable detection of NESp over both time (Fig. 1b) and freeze-thaw cycles (Fig. 1d), with negligible variation between NCC1 and NCC2. There were also fewer samples with a total loss of detection at the endpoint when using the extraction-free method compared with CE-DNA extraction (3.4% vs 7.4%, Table 2).

CE amplifies all viable gentamicin-resistant strains in a sample, the totality of which is harvested for detection [34,45]. Thus, for pneumococcal detection using CE, any pneumococcal strains present must be both viable at the time of plating and able to survive on the culture plate during incubation. In the case of saliva, this method enriches for pneumococcus alongside other gentamicin-resistant mitis-group streptococci, which all compete for resources and space [55]. Meanwhile, the extraction-free method used in this study consists simply of heat and enzymatic lysis step and direct qPCR, meaning that bacterial viability is not a requirement for detection.

Therefore, we hypothesized that the observed reduction in NCC2 strain detection following the CE-DNA extraction method was due to reduced cell survival in saliva and/or growth during CE. To test this, we compared the recovery of cells immediately after spiking into either saliva or BHI and found that while there was no difference in NESp recovery between strains when culture-enriched from BHI, recovery of NCC2 strains was significantly lower than that of NCC1 when culture-enriched from saliva (Fig. 2). These findings suggest that while all cells were viable at the time of plating, the NCC2 strains, but not NCC1, had a growth disadvantage specifically in saliva during culture enrichment. Potential explanations for this include a lower competitive fitness of these NCC2 strains against the other gentamicin-resistant bacteria present in saliva. Alternatively, these NCC2 strains may have been more susceptible to growth inhibition by a component of saliva, such as immune cells or proteases.

Previous studies have shown that *aliC* and *aliD* in NCC2 strains can compensate for the lack of capsule and aid survival in blood, by binding IgA, as well as by upregulating *cbpAC*, which increases resistance to complement deposition and neutrophil killing [24]. Additionally, these oligopeptide transporters bind peptides from other species, which can enhance carbohydrate metabolism, thus growth and colonization [56]. The presence of PspK has been shown to be necessary for NCC1 nasopharyngeal colonization in mice with a suggested role in cell adhesion similar to that of pspC [48]. Based on these findings, we hypothesized that *pspK* or *aliC* and *aliD* would also have compensatory roles in bacterial growth or survival in saliva. We found that *aliCaliD* and *pspK* knockouts both showed reduced viability compared to the wild types when spiked into BHI and tested using CE-DNA extraction. However, there were no significant differences between each mutant and wild-type strains when spiked into saliva (Fig. 2), suggesting that under these conditions and in these strains, *pspK* and *aliC*/*aliD* do not directly impact the growth or survival in saliva, and that this is determined by a more complex set of interactions.

The lack of capsule itself likely leads to reduced fitness compared with encapsulated strains, which, unlike the NCC1 strains, cannot be compensated for by NCC2 strains. A study of 57 NCC2 strains found all strains tested lacked several genes, including virulence factors pilus islet-1, *cbpA*, *pspA*, *nanE*, *glf*, *ntpK* and *rlrA* [57]. Further studies support these findings of major alterations in NCC2 genomes [19], beyond just changes in the *cps* locus, which may explain their reduced fitness during saliva-CE [8]. Further studies are needed which test the recovery of NCC1 and NCC2 strains from either heat-treated, filtered or fractionated saliva, to systematically determine the salivary component driving this apparent reduced NCC2 fitness during CE.

This study examined stability at two different bacterial loads, 1,000 and 10,000 c.f.u. ml^−1^, and the observed stability trends were generally replicated between each, with the expected ~3 Cq difference (Fig. 1). This suggests that the detection and quantification methods employed here were robust. Moreover, the recovery of representative NCC1 and NCC2 strains spiked into BHI was both highly replicable and similar between strains (Fig. 2), suggesting that the observed variations between strains could not be explained by variations in the initial quantity or viability of bacteria spiked into saliva. However, this study was limited by testing the stability of only a small number of NESp strains, and only in the group II, NCC1 and NCC2 clades. Moreover, we could not test any NCC2 strains from the MLST groups ST344 and ST448, which are predominant in conjunctivitis outbreaks [7]. Given the inter-strain differences observed here, it would be interesting to assess both additional group II NESp strains, as well as group I NESp strains. Gaining a comprehensive knowledge of the impact of capsule absence on pneumococcal viability is essential for informing future carriage study sample collection and storage practices.

NESp prevalence is likely underreported in carriage surveillance due to the limited focus on detection and characterization of these strains. We recently sought to address this by developing a method to aid the detection of NESp, particularly in oral sample types [31]. To further address the knowledge gap around NESp carriage, this current study aimed to determine the conditions for saliva collection, storage and testing for optimal NESp detection. As such, we have demonstrated that NESp can be reliably detected in saliva for up to 72 h when stored at 4 °C, or 24 h when stored at room temperature. We have also demonstrated that while glycerol supplementation of saliva slightly improves NESp detection when using CE, it is not otherwise necessary. We also showed that NCC1 strains tested here exhibited comparable resilience to encapsulated pneumococci, while NCC2 strains tested here demonstrated a competitive disadvantage during CE. Therefore, while CE may enhance detection of encapsulated pneumococci and NCC1 NESp, it can cause undue suppression of NCC2 NESp detection and possibly contribute to underreporting of NESp prevalence. Given that the extraction-free method is cheaper and less labour-intensive than CE-DNA extraction [43], it may be advisable to employ this, or other culture-free methods to ensure accurate detection of NESp. Further investigation is required to understand the mechanism underlying our observation of reduced NCC2 fitness in saliva during CE. These findings underscore the importance of refining detection methodologies and considering strain-specific differences when designing future pneumococcal carriage studies.

## Supplementary material

10.1099/mic.0.001585 Uncited Supplementary Material 1.

## Abbreviations

BHI: brain heart infusion
CbpAC: choline-binding protein AC
CE: culture enrichment
Cq: quantification cycle
NCCs: null-capsule clades
NESp: non-encapsulated *Streptococcus pneumoniae*
PCVs: pneumococcal conjugate vaccines

## References

[1] Manna S, Ortika BD, Werren JP, Pell CL, Gjuroski I (2025). *Streptococcus pneumoniae* serotype 33H: a novel serotype with frameshift mutations in the acetyltransferase gene wciG. Pneumonia.

[2] Blacklock CB, Weinberger DM, Perniciaro S, Wyllie AL (2025). Streptococcus pneumoniae serotypes. https://pneumococcalcapsules.github.io/serotypes/.

[3] Lo SW, Gladstone RA, van Tonder AJ, Lees JA, du Plessis M (2019). Pneumococcal lineages associated with serotype replacement and antibiotic resistance in childhood invasive pneumococcal disease in the post-PCV13 era: an international whole-genome sequencing study. Lancet Infect Dis.

[4] Waight PA, Andrews NJ, Ladhani SN, Sheppard CL, Slack MPE (2015). Effect of the 13-valent pneumococcal conjugate vaccine on invasive pneumococcal disease in England and Wales 4 years after its introduction: an observational cohort study. Lancet Infect Dis.

[5] Nunes S, Félix S, Valente C, Simões AS, Tavares DA (2016). The impact of private use of PCV7 in 2009 and 2010 on serotypes and antimicrobial resistance of *Streptococcus pneumoniae* carried by young children in Portugal: Comparison with data obtained since 1996 generating a 15-year study prior to PCV13 introduction. Vaccine.

[6] Sá-Leão R, Nunes S, Brito-Avô A, Frazão N, Simões AS (2009). Changes in pneumococcal serotypes and antibiotypes carried by vaccinated and unvaccinated day-care centre attendees in Portugal, a country with widespread use of the seven-valent pneumococcal conjugate vaccine. Clin Microbiol Infect.

[7] Keller LE, Robinson DA, McDaniel LS (2016). Nonencapsulated *Streptococcus pneumoniae*: emergence and pathogenesis. mBio.

[8] Iranzadeh A, Alisoltani A, Kiran AM, Breiman RF, Chaguza C (2025). Comparative pangenomics of *Streptococcus pneumoniae* from Malawi: uncovering genetic variability and pathogenicity. Microb Genom.

[9] Feldman C, Anderson R (2020). Recent advances in the epidemiology and prevention of *Streptococcus pneumoniae* infections. F1000Res.

[10] Bradshaw JL, McDaniel LS (2019). Selective pressure: rise of the nonencapsulated pneumococcus. PLoS Pathog.

[11] Takeuchi N, Ohkusu M, Hishiki H, Fujii K, Hotta M (2020). First report on multidrug-resistant non-encapsulated *Streptococcus pneumoniae* isolated from a patient with pneumonia. J Infect Chemother.

[12] Takeuchi N, Ohkusu M, Wada N, Kurosawa S, Miyabe A (2019). Molecular typing, antibiotic susceptibility, and biofilm production in nonencapsulated *Streptococcus pneumoniae* isolated from children in Japan. J Infect Chemother.

[13] Langereis JD, de Jonge MI (2017). Non-encapsulated *Streptococcus pneumoniae*, vaccination as a measure to interfere with horizontal gene transfer. Virulence.

[14] Martin CS, Bradshaw JL, Pipkins HR, McDaniel LS (2018). Pulmonary disease associated with nonencapsulated *Streptococcus pneumoniae*. Open Forum Infectious Diseases.

[15] Yokota S, Tsukamoto N, Sato T, Ohkoshi Y, Yamamoto S (2023). Serotype replacement and an increase in non-encapsulated isolates among community-acquired infections of *Streptococcus pneumoniae* during post-vaccine era in Japan. *IJID Regions*.

[16] Kawaguchiya M, Urushibara N, Aung MS, Kudo K, Ito M (2021). Clonal lineages and antimicrobial resistance of nonencapsulated *Streptococcus pneumoniae* in the post-pneumococcal conjugate vaccine era in Japan. Int J Infect Dis.

[17] Chewapreecha C, Harris SR, Croucher NJ, Turner C, Marttinen P (2014). Dense genomic sampling identifies highways of pneumococcal recombination. Nat Genet.

[18] Hathaway LJ, Stutzmann Meier P, Bättig P, Aebi S, Mühlemann K (2004). A homologue of aliB is found in the capsule region of nonencapsulated *Streptococcus pneumoniae*. J Bacteriol.

[19] Lorenz O, King AC, Hung HCH, Ganaie FA, Wyllie AL (2025). SeroBA(v2.0) and SeroBAnk: a robust genome-based serotyping scheme and comprehensive atlas of capsular diversity in *Streptococcus pneumoniae*. bioRxiv.

[20] Keller LE, Jones CV, Thornton JA, Sanders ME, Swiatlo E (2013). PspK of *Streptococcus pneumoniae* increases adherence to epithelial cells and enhances nasopharyngeal colonization. Infect Immun.

[21] Thompson CD, Bradshaw JL, Miller WS, Vidal AGJ, Vidal JE (2023). Oligopeptide transporters of nonencapsulated *Streptococcus pneumoniae* regulate *CbpAC* and *PspA* expression and reduce complement-mediated clearance. mBio.

[22] Mohale T, Wolter N, Allam M, Ndlangisa K, Crowther-Gibson P (2016). Genomic analysis of nontypeable pneumococci causing invasive pneumococcal disease in South Africa, 2003–2013. BMC Genomics.

[23] Thompson CD, Bradshaw JL, Miller WS, Vidal AGJ, Vidal JE (2023). Oligopeptide transporters of nonencapsulated *Streptococcus pneumoniae* regulate CbpAC and PspA expression and reduce complement-mediated clearance. mBio.

[24] Bradshaw JL, Pipkins HR, Keller LE, Pendarvis JK, McDaniel LS (2018). Mucosal infections and invasive potential of nonencapsulated *Streptococcus pneumoniae* are enhanced by oligopeptide binding proteins *AliC* and *AliD*. mBio.

[25] Sakatani H, Kono M, Nanushaj D, Murakami D, Takeda S (2022). A novel pneumococcal surface protein K of nonencapsulated *Streptococcus pneumoniae* promotes transmission among littermates in an infant mouse model with influenza A virus coinfection. Infect Immun.

[26] Park IH, Geno KA, Sherwood LK, Nahm MH, Beall B (2014). Population-based analysis of invasive nontypeable pneumococci reveals that most have defective capsule synthesis genes. PLoS One.

[27] Hanage WP, Kaijalainen T, Saukkoriipi A, Rickcord JL, Spratt BG (2006). A successful, diverse disease-associated lineage of nontypeable pneumococci that has lost the capsular biosynthesis locus. J Clin Microbiol.

[28] Xu Q, Kaur R, Casey JR, Sabharwal V, Pelton S (2011). Nontypeable *Streptococcus pneumoniae* as an otopathogen. Diagn Microbiol Infect Dis.

[29] Dixit C, Keller LE, Bradshaw JL, Robinson DA, Swiatlo E (2016). Nonencapsulated *Streptococcus pneumoniae* as a cause of chronic adenoiditis. IDCases.

[30] Sá-Leão R, Pinto F, Aguiar S, Nunes S, Carriço JA (2011). Analysis of invasiveness of pneumococcal serotypes and clones circulating in Portugal before widespread use of conjugate vaccines reveals heterogeneous behavior of clones expressing the same serotype. J Clin Microbiol.

[31] Laxton CS, Toekiran FL, Lin T-Y, Lomeda BD, Hislop MS (2025). An abundance of *aliC* and *aliD* genes were identified in saliva using a novel multiplex qPCR to characterize group II non-encapsulated pneumococci with improved specificity. Microbiology.

[32] Satzke C, Turner P, Virolainen-Julkunen A, Adrian PV, Antonio M (2013). Standard method for detecting upper respiratory carriage of *Streptococcus pneumoniae*: updated recommendations from the World Health Organization Pneumococcal Carriage Working Group. Vaccine.

[33] Laxton CS, Peno C, Hahn AM, Allicock OM, Perniciaro S (2023). The potential of saliva as an accessible and sensitive sample type for the detection of respiratory pathogens and host immunity. *Lancet Microbe*.

[34] Wyllie AL, Chu MLJN, Schellens MHB, van Engelsdorp Gastelaars J, Jansen MD (2014). *Streptococcus pneumoniae* in saliva of Dutch primary school children. PLoS One.

[35] Wróbel-Pawelczyk I, Ronkiewicz P, Wanke-Rytt M, Rykowska D, Górska-Kot A (2022). Pneumococcal carriage in unvaccinated children at the time of vaccine implementation into the national immunization program in Poland. Sci Rep.

[36] Wyllie AL, Rümke LW, Arp K, Bosch AATM, Bruin JP (2016). Molecular surveillance on *Streptococcus pneumoniae* carriage in non-elderly adults; little evidence for pneumococcal circulation independent from the reservoir in children. Sci Rep.

[37] Almeida ST, Paulo AC, Froes F, de Lencastre H, Sá-Leão R (2021). Dynamics of pneumococcal carriage in adults: a new look at an old paradigm. J Infect Dis.

[38] Krone CL, Wyllie AL, van Beek J, Rots NY, Oja AE (2015). Carriage of *Streptococcus pneumoniae* in aged adults with influenza-like-illness. PLoS One.

[39] Wyllie AL, Mbodj S, Thammavongsa DA, Hislop MS, Yolda-Carr D Persistence of pneumococcal carriage among older adults in the community despite COVID-19 mitigation measures. Microbiol Spectr.

[40] Allicock OM, York A, Waghela P, Yolda-Carr D, Weinberger DM (2022). Impact of temporary storage conditions on the viability of *Streptococcus pneumoniae* in saliva. mSphere.

[41] Marín-Echeverri C, Pérez-Zapata L, Álvarez-Acevedo L, Gutiérrez-Hincapié S, Adams-Parra M (2024). Diagnostic performance, stability, and acceptability of self-collected saliva without additives for SARS-CoV-2 molecular diagnosis. Eur J Clin Microbiol Infect Dis.

[42] Allicock OM, Lin T-Y, Fajardo KT, Yolda-Carr D, Hislop MS Exploring the potential of a saliva-based, RNA-extraction-free PCR test for the multiplexed detection of key respiratory pathogens.

[43] Peno C, Lin T-Y, Hislop MS, Yolda-Carr D, Farjado K (2024). A low-cost culture- and DNA extraction-free method for the molecular detection of pneumococcal carriage in saliva. Microbiol Spectr.

[44] Carvalho M da GS, Tondella ML, McCaustland K, Weidlich L, McGee L (2007). Evaluation and improvement of real-time PCR assays targeting *lytA*, *ply*, and *psaA* genes for detection of pneumococcal DNA. J Clin Microbiol.

[45] Trzciński K, Bogaert D, Wyllie A, Chu MLJN, van der Ende A (2013). Superiority of trans-oral over trans-nasal sampling in detecting *Streptococcus pneumoniae* colonization in adults. PLoS One.

[46] Wyllie AL, Pannekoek Y, Bovenkerk S, van Engelsdorp Gastelaars J, Ferwerda B (2017). Sequencing of the variable region of *rpsB* to discriminate between *Streptococcus pneumoniae* and other streptococcal species. Open Biol.

[47] Tóthpál A, Desobry K, Joshi SS, Wyllie AL, Weinberger DM (2019). Variation of growth characteristics of pneumococcus with environmental conditions. BMC Microbiol.

[48] Park IH, Kim K-H, Andrade AL, Briles DE, McDaniel LS (2012). Nontypeable pneumococci can be divided into multiple *cps* types, including one type expressing the novel gene *pspK*. mBio.

[49] Carr MA, Marcelo D, Lovell KM, Benton AH, Tullos NA (2022). Absence of *Streptococcus pneumoniae* capsule increases bacterial binding, persistence, and inflammation in corneal infection. Microorganisms.

[50] Bustin SA, Benes V, Garson JA, Hellemans J, Huggett J (2009). The MIQE guidelines: minimum information for publication of quantitative real-time PCR experiments. Clin Chem.

[51] Schaudien D, Baumgärtner W, Herden C (2007). High preservation of DNA standards diluted in 50% glycerol. Diagn Mol Pathol.

[52] Rayack EJ, Askari HM, Zirinsky E, Lapidus S, Sheikha H (2023). Routine saliva testing for SARS-CoV-2 in children: methods for partnering with community childcare centers. Front Public Health.

[53] Waghela P, Davis R, Campbell M, Datta R, Hislop MS (2025). Detection of pneumococcal carriage in asymptomatic healthcare workers. Open Forum Infect Dis.

[54] Almeida ST, Pedro T, Paulo AC, de Lencastre H, Sá-Leão R (2020). Re-evaluation of *Streptococcus pneumoniae* carriage in Portuguese elderly by qPCR increases carriage estimates and unveils an expanded pool of serotypes. Sci Rep.

[55] Okahashi N, Nakata M, Kuwata H, Kawabata S (2022). Oral mitis group streptococci: a silent majority in our oral cavity. Microbiol Immunol.

[56] Nasher F, Förster S, Yildirim EC, Grandgirard D, Leib SL (2018). Foreign peptide triggers boost in pneumococcal metabolism and growth. BMC Microbiol.

[57] Tavares DA, Simões AS, Bootsma HJ, Hermans PW, de Lencastre H (2014). Non-typeable pneumococci circulating in Portugal are of cps type NCC2 and have genomic features typical of encapsulated isolates. BMC Genomics.

[58] Keller LE, Thomas JC, Luo X, Nahm MH, McDaniel LS (2013). Draft genome sequences of five multilocus sequence types of nonencapsulated *Streptococcus pneumoniae*. Genome Announc.

