# Supplementary material for: Storage stability of non-encapsulated pneumococci in saliva is dependent on null-capsule clade, with strains carrying aliC and aliD showing a competitive disadvantage during culture enrichment
Source: Microbiology (Reading). 2025 Aug 1;171(8):001585. doi: 10.1099/mic.0.001585 (PMC12316466; doi:10.1099/mic.0.001585)
Supplement: Uncited Supplementary Material 1. [file mic-171-01585-s001.pdf]

Supplementary data: Storage stability of non-encapsulated pneumococci in saliva is dependent on null-capsule clade, with strains carrying *aliC* and *aliD* showing a competitive disadvantage during culture enrichment

Claire S. Laxton^, Orchid M. Allicock, Chikondi Peno, Tzu-Yi Lin, Alidia Koelewijn, Femke L. Toekiran, Luna Aguilar, Anna York[*], Anne L. Wyllie[*^]

^ claire.laxton@yale.edu; ^ awyllie@gmail.com

Linear regression model results tables showing the effect of time and temperature (time) or freeze-thaw cycle and supplementation (freeze thaw) on the detection of pneumococci from spiked saliva samples using the lytA adjusted Cq.

CE-DNA-extraction sample stability over temperature and time

Full model	Reference	Estimate ΔCq	P-value	Lower CI	Upper CI
TemperatureRT	4C	-0.50	0.647	-2.65	1.65
Temperature30	4C	-2.72	0.014	-4.87	-0.57
Time24	Time0	-0.84	0.346	-2.60	0.92
Time48	Time0	-0.88	0.324	-2.64	0.88
Time72	Time0	1.01	0.256	-0.74	2.77
Concentration10000	Concentration1000	-2.75	<0.001	-3.47	-2.03
NCC_2	NCC_1	5.90	<0.001	4.58	7.21
**Interactions**					
TemperatureRT:Time24	Time0, 4C	1.38	0.273	-1.10	3.87
Temperature30:Time24	Time0, 4C	5.70	<0.001	3.22	8.19
TemperatureRT:Time48	Time0, 4C	4.32	0.001	1.83	6.80
Temperature30:Time48	Time0, 4C	8.66	<0.001	6.18	11.15
TemperatureRT:Time72	Time0, 4C	4.47	0.001	1.98	6.95
Temperature30:Time72	Time0, 4C	6.39	<0.001	3.91	8.88
TemperatureRT:NCC_2	4C, NCC_1	0.75	0.429	-1.12	2.61
Temperature30:NCC_2	4C, NCC_1	4.08	<0.001	2.22	5.95

Stratifications

Stability in CE-DNA-extraction samples stored at 4C

Variable	Reference	Estimate ΔCq	P-value	Lower CI	Upper CI
Time24	Time0	-0.84	0.148	-1.99	0.31
Time48	Time0	-0.88	0.131	-2.03	0.27
Time72	Time0	1.01	0.083	-0.14	2.16
Concentration10000	Concentration1000	-3.09	<0.001	-3.91	-2.28
NCC_2	NCC_1	5.90	<0.001	5.03	6.76

Stability in CE-DNA-extraction samples stored at RT

Variable	Reference	Estimate ΔCq	P-value	Lower CI	Upper CI
Time24	Time0	0.54	0.576	-1.40	2.49
Time48	Time0	3.44	0.001	1.50	5.38
Time72	Time0	5.48	<0.001	3.54	7.42
Concentration10000	Concentration1000	-3.31	<0.001	-4.69	-1.94
NCC_2	NCC_1	6.64	<0.001	5.19	8.10

Stability in CE-DNA-extraction samples stored at 30

Variable	Reference	Estimate ΔCq	P-value	Lower CI	Upper CI
Time24	Time0	4.86	<0.001	2.76	6.97
Time48	Time0	7.79	<0.001	5.68	9.89
Time72	Time0	7.40	<0.001	5.30	9.51
Concentration10000	Concentration1000	-1.85	0.016	-3.34	-0.36
NCC_2	NCC_1	9.98	<0.001	8.40	11.56

Stability for NCC2 CE-DNA-extraction samples samples at 4C

Variable	Reference	Estimate ΔCq	P-value	Lower CI	Upper CI
Time24	Time0	-0.87	0.263	-2.42	0.69
Time48	Time0	-0.75	0.333	-2.30	0.81
Time72	Time0	1.68	0.035	0.12	3.24
Concentration10000	Concentration1000	-2.94	<0.001	-4.04	-1.84

Stability for NCC2 CE-DNA-extraction samples at RT

Variable	Reference	Estimate ΔCq	P-value	Lower CI	Upper CI
Time24	Time0	0.62	0.646	-2.10	3.33
Time48	Time0	3.92	0.006	1.20	6.64
Time72	Time0	6.70	<0.001	3.98	9.42
Concentration10000	Concentration1000	-3.38	0.001	-5.30	-1.46

Stability for NCC2 CE-DNA-extraction samples samples at 30

Variable	Reference	Estimate ΔCq	P-value	Lower CI	Upper CI
Time24	Time0	6.99	<0.001	4.38	9.60
Time48	Time0	10.05	<0.001	7.45	12.66
Time72	Time0	9.23	<0.001	6.62	11.83
Concentration10000	Concentration1000	-1.38	0.136	-3.22	0.46

Stability for NCC_1 CE-DNA-extraction samples samples at 4C

Variable	Reference	Estimate ΔCq	P-value	Lower CI	Upper CI
Time24	Time0	-0.79	0.306	-2.40	0.83
Time48	Time0	-1.14	0.148	-2.76	0.47
Time72	Time0	-0.32	0.667	-1.94	1.29
Concentration10000	Concentration1000	-3.39	<0.001	-4.53	-2.24

Stability for NCC_1 CE-DNA-extraction samples samples at RT

Variable	Reference	Estimate ΔCq	P-value	Lower CI	Upper CI
**Time24**	Time0	0.40	0.697	-1.78	2.58
**Time48**	Time0	2.48	0.029	0.30	4.66
**Time72**	Time0	3.04	0.011	0.86	5.22
**Concentration10000**	Concentration1000	-3.18	0.001	-4.72	-1.64

Stability for NCC_1 CE-DNA-extraction samples samples at 30

Variable	Reference	Estimate ΔCq	P-value	Lower CI	Upper CI
Time24	Time0	0.61	0.480	-1.22	2.44
Time48	Time0	3.25	0.002	1.42	5.08
Time72	Time0	3.76	0.001	1.93	5.59
Concentration10000	Concentration1000	-2.78	0.001	-4.08	-1.49

Extraction-free sample stability over temperature and time

Full model	Reference	Estimate ΔCq	P-value	Lower CI	Upper CI
TemperatureRT	4C	-0.91	0.265	-2.53	0.70
Temperature30	4C	-3.27	<0.001	-4.88	-1.66
Time24	Time0	0.31	0.641	-1.01	1.63
Time48	Time0	0.67	0.315	-0.65	1.99
Time72	Time0	0.50	0.452	-0.81	1.82
Concentration10000	Concentration1000	-3.28	<0.001	-3.82	-2.74
NCC_2	NCC_1	-0.38	0.453	-1.36	0.61
**Interactions**					
TemperatureRT:Time24	Time0, 4C	-1.45	0.125	-3.32	0.41
Temperature30:Time24	Time0, 4C	0.63	0.504	-1.23	2.49
TemperatureRT:Time48	Time0, 4C	-0.29	0.761	-2.15	1.58
Temperature30:Time48	Time0, 4C	4.24	<0.001	2.38	6.10
TemperatureRT:Time72	Time0, 4C	0.73	0.437	-1.13	2.60
Temperature30:Time72	Time0, 4C	5.80	<0.001	3.94	7.67
TemperatureRT:NCC_2	4C, NCC_1	1.37	0.055	-0.03	2.77
Temperature30:NCC_2	4C, NCC_1	4.90	<0.001	3.51	6.30

Stratifications

Stability in extraction-free samples stored at 4C

Variable	Reference	Estimate ΔCq	P-value	Lower CI	Upper CI
Time24	Time0	0.31	0.340	-0.34	0.96
Time48	Time0	0.67	0.043	0.02	1.32
Time72	Time0	0.50	0.127	-0.15	1.15
Concentration10000	Concentration1000	-3.21	<0.001	-3.67	-2.75
NCC_2	NCC_1	-0.38	0.127	-0.86	0.11

Stability in extraction-free samples stored at RT

Variable	Reference	Estimate ΔCq	P-value	Lower CI	Upper CI
Time24	Time0	-1.14	0.017	-2.07	-0.21
Time48	Time0	0.39	0.406	-0.54	1.31
Time72	Time0	1.24	0.010	0.31	2.16
Concentration10000	Concentration1000	-3.36	<0.001	-4.02	-2.71
NCC_2	NCC_1	0.99	0.006	0.30	1.69

Stability in extraction-free samples stored at 30

Variable	Reference	Estimate ΔCq	P-value	Lower CI	Upper CI
Time24	Time0	0.94	0.360	-1.11	2.99
Time48	Time0	4.91	<0.001	2.86	6.97
Time72	Time0	6.31	<0.001	4.25	8.36
Concentration10000	Concentration1000	-3.27	<0.001	-4.72	-1.82
NCC_2	NCC_1	4.53	<0.001	2.99	6.07

Stability for NCC2 extraction-free samples at 4C

Variable	Reference	Estimate ΔCq	P-value	Lower CI	Upper CI
Time24	Time0	0.40	0.329	-0.42	1.22
Time48	Time0	0.45	0.272	-0.37	1.27
Time72	Time0	0.53	0.195	-0.29	1.35
Concentration10000	Concentration1000	-3.32	<0.001	-3.90	-2.74

Stability for NCC2 extraction-free samples at RT

Variable	Reference	Estimate ΔCq	P-value	Lower CI	Upper CI
Time24	Time0	-0.57	0.311	-1.71	0.56
Time48	Time0	0.77	0.178	-0.37	1.90
Time72	Time0	2.00	0.001	0.87	3.14
Concentration10000	Concentration1000	-3.58	<0.001	-4.39	-2.78

Stability for NCC2 extraction-free samples at 30

Variable	Reference	Estimate ΔCq	P-value	Lower CI	Upper CI
Time24	Time0	2.88	0.027	0.35	5.40
Time48	Time0	7.21	<0.001	4.69	9.74
Time72	Time0	8.50	<0.001	5.97	11.02
Concentration10000	Concentration1000	-3.08	0.001	-4.86	-1.29

Stability for NCC_1 extraction-free samples at 4C

Variable	Reference	Estimate ΔCq	P-value	Lower CI	Upper CI
Time24	Time0	0.14	0.814	-1.13	1.41
Time48	Time0	1.12	0.078	-0.15	2.39
Time72	Time0	0.45	0.455	-0.82	1.71
Concentration10000	Concentration1000	-2.99	<0.001	-3.88	-2.09

Stability for NCC_1 extraction-free samples at RT

Variable	Reference	Estimate ΔCq	P-value	Lower CI	Upper CI
**Time24**	Time0	-2.28	0.009	-3.85	-0.71
**Time48**	Time0	-0.38	0.610	-1.95	1.20
**Time72**	Time0	-0.30	0.687	-1.87	1.28
**Concentration10000**	Concentration1000	-2.92	<0.001	-4.03	-1.80

Stability for NCC_1 extraction-free samples at 30

Variable	Reference	Estimate ΔCq	P-value	Lower CI	Upper CI
Time24	Time0	-2.93	0.002	-4.55	-1.30
Time48	Time0	0.32	0.678	-1.31	1.94
Time72	Time0	1.92	0.025	0.29	3.54
Concentration10000	Concentration1000	-3.65	<0.001	-4.80	-2.50

CE-DNA-extraction sample stability over freeze thaw

Full model	Reference	Estimate ΔCq	P-value	Lower CI	Upper CI
Temperature-20	-80C	0.00	1.000	-1.96	1.96
ft_cycle1	ft_cycle0	0.58	0.559	-1.38	2.55
ft_cycle2	ft_cycle0	1.64	0.101	-0.32	3.61
ft_cycle3	ft_cycle0	2.34	0.020	0.37	4.30
Storageraw	gly	4.50	<0.001	3.52	5.48
Concentration10000	Concentration1000	-3.50	<0.001	-4.48	-2.52
NCC_2	NCC_1	4.21	<0.001	3.17	5.25
**Interactions**					
ft_cycle1:NCC_2	ft_cycle0, NCC1	1.76	0.272	-1.39	4.90
ft_cycle2:NCC_2	ft_cycle0, NCC1	0.81	0.584	-2.12	3.75
ft_cycle3:NCC_2	ft_cycle0, NCC1	0.42	0.778	-2.51	3.35
Storageraw:ft_cycle1	ft_cycle0, gly	-0.77	0.588	-3.55	2.02
Storageraw:ft_cycle2	ft_cycle0, gly	-0.81	0.567	-3.59	1.97
Storageraw:ft_cycle3	ft_cycle0, gly	1.62	0.251	-1.16	4.41

Stability in CE-DNA-extraction samples stored at -80

Full model	Reference	Estimate ΔCq	P-value	Lower CI	Upper CI
Storageraw	gly	3.79	<0.001	2.58	5.00
ft_cycle1	ft_cycle0	0.58	0.501	-1.13	2.30
ft_cycle2	ft_cycle0	1.64	0.060	-0.07	3.36
ft_cycle3	ft_cycle0	2.34	0.008	0.62	4.05
Concentration10000	Concentration1000	-3.16	<0.001	-4.38	-1.95
NCC_2	NCC_1	4.38	<0.001	3.09	5.66

Stability in CE-DNA-extraction samples stored at -20

Full model	Reference	Estimate ΔCq	P-value	Lower CI	Upper CI
Storageraw	gly	5.21	<0.001	3.65	6.78
ft_cycle1	ft_cycle0	1.53	0.174	-0.69	3.74
ft_cycle2	ft_cycle0	4.46	<0.001	2.25	6.68
ft_cycle3	ft_cycle0	4.39	<0.001	2.17	6.60
Concentration10000	Concentration1000	-3.83	<0.001	-5.40	-2.26
NCC_22	NCC_1	4.04	<0.001	2.38	5.70

Extraction-free sample stability over freeze thaw

Full model	Reference	Estimate ΔCq	P-value	Lower CI	Upper CI
Temperature-20	-80C	0.12	0.507	-0.23	0.47
ft_cycle1	ft_cycle0	-0.16	0.500	-0.64	0.32
ft_cycle2	ft_cycle0	-0.03	0.921	-0.53	0.48
ft_cycle3	ft_cycle0	0.47	0.058	-0.02	0.95
Storageraw	gly	-2.22	<0.001	-2.57	-1.87
Concentration10000	Concentration1000	-3.02	<0.001	-3.36	-2.67
NCC_2	NCC_1	-0.14	0.470	-0.51	0.23

CE-DNA-extraction refers to samples which were culture-enriched then underwent DNA extraction before qPCR. Extraction-free refers to samples which were not culture-enriched, instead undegoing a heat-enzymatic lysis step before direct-qPCR. P<0.05 was considered signficant, and those values are highlighted yellow.
